# Prevalence and incidence of external genital warts in a sample of Italian general female population

**DOI:** 10.1186/s12879-017-2202-6

**Published:** 2017-02-06

**Authors:** Barbara Suligoi, Giorgio Vittori, Maria Cristina Salfa, Laura Timelli, Dario Corsini, Giovanni Fattorini, Luciano Mariani, the Genital Warts 2 (GW2) Working Group, Daniele Agostinelli, Antonio Salvatore Amorosi, Luisa Barbaro, Rosetta Barretta, Fiorella Bencivenga, Maria Grazia Castiglione, Stefania Cinquerrui, Maddalena Cipolletta, Francesca Como, Concetta Corallo, Bianca Rosa Cozza, Marco Danna, Nicoletta Danuso, Barbara Del Bravo, Maddalena Di Noia, Natalino Ferrara, Grazia Fischetti, Gennaro Forcella, Patrizia Franzolini, Valentina Garozzo, Chiara Antonia Genco, Maristella Ghiazza, Rosa Guagliardo, Sonja Jazbec, Alessandra Lui, Sara Mantegazza, Annamaria Marcazzò, Oscar Martinelli, Claudia Mazzei, Raffaella Mezzopane, Daniela Minorini, Francesco Montoneri, Sabina Oldani, Maurizio Orlandella, Daniela Palano, Rosanna Palmiotto, Rosalba Percuoco, Maria Pizza, Carmela Placco, Adriana Santoro, Antonio Scanu, Maria Sprecaviscole, Marina Toschi, Annamaria Voltolini

**Affiliations:** 1 Centro Operativo AIDS, Istituto Superiore di Sanità, Viale Regina Elena 299, Rome, Italy; 2 Ospedale San Carlo di Nancy, Via Aurelia 275, Rome, Italy; 3 Informa srl, Via Luigi Rava 43, Rome, Italy; 4 Associazione ginecologi territoriali (AGITE), Via G. Abamonti 1, Milan, Italy; 5 HPV-unit, Istituto Nazionale Tumori Regina Elena, Via Elio Chianesi 53, Rome, Italy

**Keywords:** External genital warts, Women, Community gynecologists, Prevalence, Incidence, Italy

## Abstract

**Background:**

The Human papillomavirus is the most common sexually transmitted virus worldwide. The objective of this study was to estimate: 1) the prevalence and the incidence of external genital warts (eGW) in a sample of women attending community outpatient clinics and 2) the total number of eGW cases in the Italian female population aged 15–64 years.

**Methods:**

A prospective study was performed for a 12-month period between 2009 and 2010, among a sample of women attending community gynecological outpatient clinics located throughout Italy. Demographic data, for every woman aged 15–64 years, were collected. For women diagnosed with eGW, behavioral and clinical data were recorded. Prevalence of eGW was calculated as the proportion between the number of women with eGW and that of women visiting any of the participating gynecologists; incidence of eGW was calculated as the proportion between the number of women with a new diagnosis of eGW and that of women visiting any of the participating gynecologists. Standardized prevalence by age was used to estimate the number of eGW cases occurring in the Italian female population aged 15–64 years.

**Results:**

In 2009–2010, 44 community gynecologists were included in the network. In one-year period, 16,410 women visited any of the participating gynecologists; 63 women were diagnosed with eGW, corresponding to a prevalence of 3.8 cases per 1,000 women per year (95%CI: 2.9-4.9). The incidence of eGW was 3.0 cases per 1,000 women per year (95%CI: 2.2-3.9). Women aged 15–24 years showed both the highest prevalence and incidence. Prevalence and incidence significantly decreased by increasing age group (*p* <0.001), and were higher in Southern Italy compared to Central-Northern Italy. The estimated number of women with eGW among women aged 15–64 years in Italy, in 2010, was approximately 69,000.

**Conclusions:**

These data show a high prevalence and incidence of eGW among young women in Italy, stress the effectiveness of community clinical networks in investigating STI epidemiology among women from the general population, confirm the relevance of HPV vaccination programs among adolescents, and underscore the need of promoting safe sex, implementing early diagnosis, treatment and prevention of genital warts.

## Background

The Human papillomavirus (HPV) is the most common sexually transmitted virus worldwide. Among the more than 200 different types of HPV identified so far, more than 40 types are responsible of infections in the genital area, causing both benign and malignant lesions [1–3]. The most common benign genital HPV infection are genital warts, caused in about 90% of the cases by HPV type 6 and 11 [4, 5]. Genital warts affect both males and females; in Europe, there is an approximate burden of 500,000 cases divided almost equally between both sexes, although slightly higher in men according to latest data [6].

In Europe, genital warts are subject to mandatory notification only in the United Kingdom [7]. In other countries, the information on the spread of this disease is derived from epidemiological studies, sales of medicines specifically used for the treatment of genital warts, or surveillance of sexually transmitted infections (STI) [6].

In Italy, women with external genital warts (eGW) are usually diagnosed and treated in gynecological services and sexually transmitted infection (STI) clinics. The sentinel STI surveillance system based on STI clinics and two epidemiological studies provided data on eGW in Italy so far [8–10]. However, information obtained from the sentinel STI surveillance system, although covering 24 years of data collection and being essential for monitoring trends in time, is not suitable for estimating prevalence and incidence of single STIs in that denominators for calculating these measures are not available. Moreover, STI clinics collect data on symptomatic patients who are not representative of the population at large [8]. In Italy, about 66% of women between 18 and 55 years of age undergo every year a gynecological visit in a public and/or private gynecological setting [11].

The launch of the HPV vaccination campaign in Italy in 2008 emphasized the debate about the cost/benefit of HPV vaccination at National level and underscored the lack of prevalence/incidence data on genital warts that would be valuable as first-line efficacy indicators of the vaccination campaign.

Taking into account this background, the Istituto Superiore di Sanità (ISS) and the Italian Society of Gynecology and Obstetrics (SIGO) decided in 2009 to conduct a prospective study to estimate the prevalence and the incidence of eGW in a sample of women attending community gynecological outpatient clinics and the total number of women with eGW among women aged 15–64 years in Italy.

## Methods

### Study design and sampling method

A prospective study was performed for a 12-month period between November 2009 and December 2010, in a sample of women attending community gynecological outpatient clinics located throughout Italy (i.e. North, Center, and South).

The sample selection was based on a two sampling stage strategy: first, the selection of community gynecologists and second, the number of women visited in one year by each community gynecologist.

### The network of community gynecologists

SIGO together with the support of the Italian Association of Territorial Gynecologists (AGITE) in the first phase identified 30 gynecologists to act as regional coordinators, including at least one coordinator from each of the 20 Italian regions. The study coordinators trained the regional coordinators on the study methods and data collection procedures.

In the second phase, a list of 485 community gynecologists was obtained from the 30 lists provided by the regional coordinators. From this list, 320 community gynecologists were randomly selected and invited to participate in the study. Of these, 44 accepted to participate in the study. The regional coordinators trained the 44 community gynecologists who were residing in 13 of the 20 Italian regions.

As previously described [12], to be part of the network, the community gynecologists needed to comply with the following requirements:

- to dedicate part of their professional practice to first-level consultations (they see new patients and do the first diagnosis of the disease, they are the first doctors/physician the patients seeks for help) or referral consultations (patients are referred from other specialists for diagnosis confirmation and treatment), in either private or public practice;
- to possess a personal computer with internet connection;
- to have at least 200 women visiting the clinic in one year;
- to participate without any economic benefit.

### Sample size calculation

Standard statistical methods used for sample size estimate and analysis do not include the between-clusters component of variability in the outcome and consequently cannot be applied in cluster-based studies because they would provide sample sizes that are too small [13]. In fact, in this case the variability of the studied phenomenon depends on two components: the variability within the cluster (i.e. difference in the frequency of eGW diagnosis between women visited by the same community gynecologist) and the variability between clusters (i.e. differences between women visiting different community gynecologists). The within-cluster and between-clusters components of variability can be combined in a single statistical measure of between-clusters heterogeneity (or within-cluster homogeneity), namely the Intraclass correlation coefficient (ICC).

As previously described [12], to calculate the sample size, the following parameters were taken into account:

- the average number of women visiting any community gynecologist in one year;
- the expected incidence of eGW;
- the ICC.

Particularly, we assumed that the incidence of eGW in Italy was expected between 1 and 5 per 1,000 [9]. No estimate of the expected ICC in studies performed among Italian gynecologists was available; therefore, we used several estimates of ICC obtained from 16 datasets covering a range of diseases for health binary outcomes [9, 14].

Finally, we performed a simulation varying the median number of women visiting the same community gynecologist, the expected ICC, and the width of the 95% confidence interval (95%CI) around the expected incidence of eGW. We observed that the number of community gynecologists to be included and, consequently, the number of women included increased with increasing ICC (independently from the median number of women visited per community gynecologist), and conversely decreased with increasing 95%CI width (regardless of the ICC).

### Inclusion and exclusion criteria

Women meeting the following inclusion criteria were included [12]:

- age from 15 to 64 years;
- permanent residence in Italy (regardless of nationality);
- visited the gynecologist at least once in a one-year period.
- The exclusion criteria were:
- not having a permanent residence in Italy.

### Definition of eGW

According to Cartier’s definition [15], eGW were defined as flesh-colored, exophytic lesions (small bumps, flat, verrucous, peduncolated, raised papules or dome-shaped lesions on keratinized skin) on the external genitalia, including vulva, perineum, and perianal skin. The diagnosis of eGW was based on visual inspection. In order to standardize inter-observer variation in the diagnosis of eGW and HPV-related lesions a two-day special training course was held.

Newly diagnosed cases of eGW included women diagnosed with eGW who reported never having had eGW before.

Early recurrent cases of eGW (recent relapse) included women who reported episodes of eGW in the previous 12 months.

Late recurrent cases of eGW (late relapse) included women who reported prior episodes of eGW but did not suffer from any recurrence in the previous 12 months.

### Data collected

Participating gynecologists reported essential individual socio-demographic data (i.e. age, nationality, and ethnicity) and medical history (i.e. self-reported anti-HPV vaccination status; vaccination with bivalent or quadrivalent vaccine; number of pregnancies) for every woman aged 15–64 visiting the gynecologist for any reason.

For women diagnosed with eGW the following additional information was recorded: level of education, self-reported behavioral data (age at first sexual intercourse, previous STI lifetime, concurrent diseases, use of contraceptives in the previous 12 months, number of partners in the previous 12 months), clinical data (current genitourinary symptoms such as itching, vaginal discharge, dispareunia), written result of HIV test, reason of the consultation, type of medical or surgical treatment of eGW and outcome of the treatment.

### Data input

The collection and management of data were made electronically through the SIGO website. Each community gynecologist accessed the dedicated area containing data collection forms. Data collection forms filled by every community gynecologist were conveyed at the Contract Research Organization (CRO) data center where data management and analyses were performed. A tailored data collection software performed consistency and completeness checks on the data. The CRO performed periodical controls on the congruency and completeness of data entered by gynecologists.

### Measures of frequency

Prevalence of eGW was calculated as the proportion between the number of women with eGW and that of women visiting any of the participating gynecologists for any reason in 2009–2010. Incidence of eGW was calculated as the proportion between the number of women with a new diagnosis of eGW and that of women visiting any of the participating gynecologists for any reason in 2009–2010.

Ninety five percent confidence intervals (95%CI) were calculated using binomial distribution. Prevalence and incidence were also analyzed stratifying by age group (15–24, 25–34, 35–44, 45–64 years) and geographical area of residence (Northern, Central, Southern Italy). Comparisons between age groups or geographical areas were performed using the chi-square test. Age-specific rates were used to calculate standardized prevalence and incidence using as reference Italian [16] and European standard population [17] aged 15–64 years. Age-specific rates were used also to estimate the number of eGW cases in the Italian female population aged 15–64 years multiplying the estimated prevalence by the underlying female population. For the analysis of data, SaS.8.2 was used.

## Results

In one-year period, 16,410 women, who represented 0.1% of the total female Italian population aged 15–64 years, visited any of the 44 community gynecologists included in the network. Their median age was 37 years (inter quartile range 29–47 years); 10.5% were pregnant; 12.5% were migrants: of these, 56.0% were Caucasians, 17.0% Hispanic, 15.0% Asian, 11.0% African and 1.0% of other ethnicities.

Fifty women had been vaccinated for HPV; of these, 30 with the bivalent vaccine, 9 with the quadrivalent vaccine, and the type of vaccine was not reported for 11 women.

There were 63 women diagnosed with eGW. Socio-demographic and behavioral characteristics of these women are shown in Table 1. The median age at first sexual intercourse was 17 years (inter quartile range: 15–20 years). Ten women (16.0%) reported having had previous bacterial genital infections without specifying the etiological agent; one woman reported having had genital herpes, and one *Chlamydia trachomatis* infection.

**Table 1:** Socio-demographic and behavioral characteristics of 63 women with eGW

Characteristic	Women with eGW (*n* = 63)	
	*N*	%
Education		
None		
Primary school	12	19.1
Middle school	9	14.3
High school	25	39.7
University degree	17	26.9
na°		
Current pregnancy		
yes	7	11.1
no	56	88.9
na°		
Number of sexual partners in previous 12 months		
0	26	41.3
1	30	47.6
≥ 2	7	11.1
na°		
Current genitourinary symptoms		
yes	32	50.8
no	31	49.2
na°		
Contraceptive use in previous 12 months		
None		
Condom	16	25.4
Oral contraceptive	11	17.5
Other	13	20.6
na°		35.5

°na: not available

Moreover, 13.0% of women with eGW reported having been tested for HIV at least once in a lifetime; none of them was HIV positive. No woman with eGW had been HPV-vaccinated.

Among women with eGW, the most frequent reasons for consultation were as follows: presence of genital warts (36.0%), check-up not related to eGW (35.0%; includes opportunistic cytology screening, annual check-up, post-treatment follow-up, family planning), self-reported genital symptoms (11.0%), pregnancy (10.0%), menopause (5.0%), and use of oral contraceptives (5.0%) (these categories were not mutually exclusive).

The crude prevalence of eGW was 3.8 cases per 1,000 women per year (95%CI: 2.9-4.9) (Table 2).

**Table 2:** Crude prevalence of eGW, by age group and location (per 1,000)

Age group	Women with eGW (number)	Women visiting the gynecologist (number)	Prevalence (‰) (95% CI)			
			Total	North	Center	South
15–24	18	2,491	7.2 (4.3-11.4)	8.3 (4.2-14.9)	0.0 (0–38.8)	6.5(1.6-13.9)
25–34	27	4,331	6.2 (4.1-9.1)	2.6 (1.0-5.7)	3.8 (0.1-21.1)	11.3 (6.9-17.5)
35–44	11	4,482	2.5 (1.2-4.4)	0.8 (0.1-3.0)	0.0 (0–15.8)	8.3 (4.2-14.9)
45–64	7	5,106	1.4 (0.6-2.8)	8.3 (4.2-14.9)	0.0 (0–13.9)	4.9 (2.3-9.4)
Total	63	16,410	3.8 (2.9-4.9)	2.6 (1.6-4.0)	1.2 (0.0-6.5)	5.4 (3.9-7.3)

*p* < 0.001 by age group; *p* = 0.003 North + Center vs. South

The highest prevalence of eGW was observed among 25–34 year-old women living in the South. Prevalence decreased by increasing age group: this decrease was significant even though 95%CI overlapped (*p* <0.001) [18] (Table 2). When combining data of women living in the North with those of women living in the Center (to reach statistical significance), the prevalence of eGW was significantly higher among women living in the South (5.4‰ vs. 2.5‰, *p* = 0.003). The prevalence of eGW among Italian and migrant women was not significantly different (3.9‰ vs. 3.4‰, *p* = 0.733).

Among the 63 women diagnosed with eGW, 49 were new diagnoses corresponding to a crude incidence of 3.0 cases per 1,000 women per year (95%CI: 2.2-3.9) (Table 3). Eight women had a recurrent eGW; of these, 4 had a late relapse and 4 had a recent relapse (corresponding in both cases to an incidence of 0.2 cases per 1,000 women per year, 95%CI: 0.1-0.6). For 6 women this clinical information was not reported.

**Table 3:** Crude incidence of eGW, by age group and location (per 1,000)

Age group	Women with eGW (number)	Women visiting the gynecologist (number)	Incidence (‰) (95% CI)			
			Total	North	Central	South
15–24	15	2,491	6.0 (4.3-11.4)	6.8 (3.1-13.0)	0.0 (0–38.8)	5.6 (2.0-12.1)
25–34	21	4,331	4.8 (4.1-9.1)	2.1 (0.7-5.1)	3.8 (0.1-21.1)	8.5 (4.8-14.0)
35–44	8	4,482	1.8 (1.2-4.4)	0.8 (0.1-3.0)	0.0 (0–15.8)	3.3 (1.2-7.1)
45–64	5	5,106	1.0 (0.6-2.8)	8.3 (4.2-14.9)	0.0 (0–13.9)	1.3 (0.3-3.8)
Total	49	16,410	3.0 (2.2-3.9)	2.3 (1.3-3.6)	1.2 (0.0-6.5)	3.9 (2.7-5.6)

*p* < 0.001 by age group; *p* = 0.036 North + Center vs. South

The crude incidence of eGW significantly decreased by increasing age group (*p* <0.001). The highest incidence was observed among 25–34 year-old women living in the South and 45–64 year-old women living in the North. The incidence of eGW was higher among women living in Southern Italy compared to those living in Central-Northern Italy (3.9‰ vs 2.2‰, *p* = 0.036). The incidence of eGW among Italian and migrant women was not significantly different (3.4‰ vs 2.9‰, *p* = 0.710).

The age-standardized prevalence and incidence of eGW using Italian female population as reference were 3.4 (95% CI: 2.6-4.3) and 2.7 (95% CI: 1.9-3.4) cases per 1,000 women aged 15–64 years, respectively. Using the European female standard population as reference, these measures were 3.6 (95% CI: 2.7-4.5) and 2.8 (95% CI: 2.0-3.6), respectively.

Using the age-specific rates of the Italian female population, we estimated that there were about 69,000 women with eGW aged 15–64 years in Italy, in 2010.

## Discussion

This prospective study estimated the prevalence and the incidence of eGW in a sample of women attending community gynecology outpatient clinics in Italy.

These data are similar to those reported in a retrospective Italian study conducted among community gynecologists [9].

The prevalence of eGW estimated in the present study (3.8 cases per 1,000) is slightly higher compared to that reported in studies conducted in other European countries in various clinical settings. In England, the prevalence of eGW was 2.3 cases per 1,000 in a sample of women attending genitourinary medicine clinics [13]. In France, it was 2.3 cases per 1,000 in a sample of women attending French public gynecologists [14]. In Spain, it was 1.6 cases per 1,000 women [19] and in Germany 1.5 cases per 1,000 women [20] attending various medical specialists. In Canada, it was 1.4 per 1,000 women based on data collected by STI clinics, pharmacists, physicians billing database and hospitalizations [21]. The prevalence found in the present study was higher also when compared to that emerged in studies based on women attending general practitioners (GP), which ranged between 0.51 and 0.59 cases per 1,000 women [10, 13, 22]. At least in the Italian context, the network of community gynecologists is likely to be more accurate in estimating the frequency of eGW among women than the GP network. The reason being that in Italy women with eGW primarily seek treatment with a gynecologist rather than a GP [10], similarly to what reported in England where about 70% of patients with eGW were seen only in genitourinary medicine clinics [13].

Conversely, the eGW prevalence estimated in this study was much lower than that reported in a retrospective study based on a self-administered questionnaire, conducted in 70,000 women of four northern European countries (Norway, Sweden, Denmark and Iceland) which reported a prevalence of 13 cases per 1,000 women [23]. Indeed, retrospective studies based on self-administered questionnaire may overestimate the frequency of GW due to a methodological bias, in that women with a history of GW would be more prone to answer to the questionnaire.

The incidence of eGW observed in this study is similar to that reported in other studies conducted in France [14] and the UK [22], but higher compared to that reported in other European countries, such as Spain [19] and Germany [20].

Compared to estimates reported in a systematic review [24], our prevalence and incidence rates are higher than those reported in other countries. These diversities can be attributed to a number of reasons, such as: a different age range of the study population (15–64 years in our sample vs. larger age ranges in other studies), a different background of the study population (women visiting a community gynecologist in our study vs. general population or privately-insured women in other studies), or a different recruitment setting (community gynecologists in our study vs. cytology screening services or GP in other studies).

We observed the highest prevalence and incidence of eGW was observed among 15–24 year-old women compared to women older than 25 years of age, similarly to what reported in other studies [14, 20–22, 24]. This finding has been associated with higher levels of sexual activity with multiple partners and low viral immunity in this age group [2]. Previous studies conducted in Italy have shown the determinant role of multiple sex partners in the prevalence of genital HPV infection among young women [25, 26], thus underscoring the main relevance of HPV vaccination among adolescent females. Interestingly, our results show a second peak in prevalence and incidence among women aged 45–64 years living in the North; this U-shaped curve has been reported in Southern Europe and may be related to immuno-senescence, perimenopausal hormonal changes, changes in male/female sexual behavior, cohort effects, or higher rates of HPV persistence at older ages [27].

Compared to the general female Italian population [16], our sample was younger (age group 25–34 years: 26.4% vs 19.2%), with a higher proportion of migrant (12.5% vs 8.5%) and pregnant women (10.5% vs 1.6%). Age-specific standardization was applied to adjust prevalence and incidence rates for differences in age distribution. The higher proportion of migrant or pregnant women in our study sample may have had an impact on the observed prevalence in that both populations have been reported to have a higher probability of HPV infection compared to Italian or non-pregnant women [28, 29]. Moreover, previous studies have evidenced a higher HPV prevalence among migrant women in Southern Italy [29, 30], which can explain the higher prevalence and incidence of eGW found among women living in the South.

In Italy, the national HPV vaccination campaign started in 2008 and targeted 12 years-old girls [31]. Our study was conducted in the two years following the implementation of the HPV vaccination program and included women aged ≥15 years which did not allow detecting any impact of the vaccination campaign on study participants. Nevertheless, our results provide essential epidemiological data that can be used as baseline for future studies aimed at evaluating the effectiveness of the Italian HPV vaccination campaign. Our study sample included a number of HPV-vaccinated women and none of them was diagnosed with eGW: these women were not comprised in the vaccination campaign but rather purchased the vaccine on their own.

Our estimated number of about 69,000 women with eGW aged 15–64 years in Italy in 2010, would imply that our sample is representative of all Italian women and that all women visit a gynecologist routinely. However, we know that around 66% of Italian women aged 18–55 years visit a gynecologist annually [11]. Therefore, assuming that women who do not visit a gynecologist annually be free from eGW, then an adjusted estimate of the total number of women aged 15–64 with eGW would be approximately 45,500 (i.e., 69,000 x 0.66). This figure should be considered as a minimum estimate, which implies that among the 34% of women who do not visit a gynecologist none is suffering from eGW.

Some limitations of this study should be addressed. The main limitation was the small number of community gynecologists who accepted to participate, which was probably attributable to the free and unpaid participation in the network. Another limitation was the lack of geographic representativeness of the participating gynecologists that resided in only 13 of the 20 Italian regions. Finally, our network of community gynecologists, though composed of generalist gynecologists, may have concentrated a population at higher risk of eGW in the study period due to the established general experience of these gynecologists.

Our surveillance network of community gynecologists shows several strengths. First, a low cost as the participation of gynecologists in the network was voluntary without any economic compensation. Second, women enrolled in the study were not selected by the presence of specific symptoms and included both symptomatic and asymptomatic patients attending for a routine visit or annual check-up, unlike STI specialists that see mainly symptomatic patients. Third, participating gynecologists were adequately trained on genital warts and could provide reliable diagnosis and follow-up of patients. Fourth, community gynecologists collected essential data on the number and the characteristics of visited women, providing the denominator required for estimating the burden of eGW in terms of frequency, incidence and prevalence. Fifth, the user-friendly data-collection software facilitated input and linkability of patient’s longitudinal data, allowing also for real-time transmission of data. Sixth, all information was centralized thus optimizing data management and analysis.

## Conclusions

This prospective study was based on an innovative surveillance network composed of a sample of community gynecologists located in 13 Italian regions.

These data stress the effectiveness of community clinical networks in investigating STI epidemiology among women from the general population, promoting safe sex and implementing early diagnosis, treatment and prevention. Moreover, this study provides evidence of the paramount importance of HPV vaccination among adolescents, which has already proved to reduce dramatically the incidence of GW in several countries [32]. Indeed, although GW represent a benign condition, treatment costs and impact on psycho-sexual life enforce the need of preventing the occurrence of these lesions.

## Abbreviations

AGITE: Italian association of territorial gynaecologistsGynecologists
CRO: The contract research organization
eGW: External genital warts
GP: General practitioners
GW: Genital warts
HPV: Human papillomavirus
ICC: Intraclass correlation coefficient
ISS: Istituto superiore di sanità
SIGO: The Italian society of gynaecology and obstetrics
STI: Sexually transmitted infections
